# Supplementary material for: Hikikomori: A Scientometric Review of 20 Years of Research
Source: Int J Environ Res Public Health. 2023 Apr 27;20(9):5657. doi: 10.3390/ijerph20095657 (PMC10177810; doi:10.3390/ijerph20095657)
Supplement: Supplementary file 1 [file ijerph-20-05657-s001.zip › ijerph-2201296-supplementary.pdf]

## Supplementary Results

### 1. Results with CiteSpace version 6.1.R2

#### *1.1. Structural Metrics*

The final optimised network obtained from the DCA consisted of 911 nodes with 3494 links, which indicates an average of 3.84 connections with other references for each node. The network had a modularity-Q index of 0.896 and a mean silhouette score of 0.950, indicating high divisibility of the network into homogeneous clusters.

#### *1.2. Thematic Clusters*

A total of 14 major clusters were identified in the final optimised network (Table S1). The largest cluster #0 consisted of 105 nodes and had a silhouette score of 0.95, with the constituent references being published in 2019 on average. The cluster was manually labelled “Risk factors and etiology”. Second, cluster #1 consisted of 97 nodes and had a silhouette score of 0.895, with the constituent references being published in 2019 on average. The cluster was manually labelled ”Clinical features”. Third, cluster #3 consisted of 44 nodes and had a silhouette score of 0.978, with the constituent references being published in 2011 on average. The cluster was manually labelled ”Family factors”. Clusters #3, #10 and #6 had the oldest mean year of publication (2011, 2012 and 2013 respectively) whereas Clusters #0, #1 and #13, #19 and #20 were the most recent clusters with a mean year of publication in 2019.

**Table S1.** Metrics of the 14 clusters identified with the DCA. Log-likelihood Ratio (LLR) labels are automatically generated by the software.

| Cluster ID | Size | Silhouette | Mean Publication Year | LLR Label | Suggested Label |
|---|---|---|---|---|---|
| 0 | 105 | 0.950 | 2019 | Office Worker | Risk factors and etiology |
| 1 | 97 | 0.895 | 2019 | Psychometric properties | Clinical features |
| 3 | 44 | 0.978 | 2011 | Strategic foundation | Family factors |
| 5 | 31 | 0.907 | 2014 | Social withdrawal behaviour | Social withdrawal |
| 6 | 30 | 0.988 | 2013 | Hong Kong | Youth services |
| 8 | 21 | 1.000 | 2016 | Virtual world | Censure and empowerment of hikikomori |
| 9 | 21 | 0.929 | 2017 | Willful subject | Sociological perspective of hikikomori |
| 10 | 20 | 0.97 | 2012 | Transcending label | Japanese youth labels |
| 13 | 19 | 0.993 | 2019 | Cultural idiom | Hikikomori across cultures |
| 19 | 15 | 1.000 | 2019 | Recommendation | Social media use |
| 20 | 15 | 0.992 | 2019 | Parent | Internet use |
| 28 | 9 | 0.992 | 2018 | Severe social withdrawal syndrome | Biological markers |
| 29 | 9 | 1.000 | 2017 | Preventing hikikomori | Gaming as an intervention for hikikomori |
| 34 | 7 | 0.988 | 2015 | Young generation | Social behaviours |

#### *1.3. Citation Burstness*

A total of 14 documents exhibited a citation burst (Table S2), after duplicates of the same documents were omitted. Out of these 14 documents, 5 of them belong to cluster #0, 6 to cluster #1, 1 to cluster #5, 1 to cluster #29. The article with the strongest citation burst was authored by Teo [12] with a score of 6.74, with the burst beginning in 2013 to 2018. There were 3 articles with the longest burst duration of 5 years: Teo [12], Teo and Gaw [14] from 2013 to 2018, and Saito and Angles [10] from 2017 to 2022. The article with the highest sigma value of 1.36 was authored by Yong and Nomura [15].

**Table S2.** Top 14 publications in terms of burst strength.

| Reference | Citation Burstness | Publication Year | Burst Begin | Burst End | Duration | Betweenness Centrality | Sigma |
|---|---|---|---|---|---|---|---|
| Teo [12] | 6.74 | 2010 | 2013 | 2018 | 5 | 0.00 | 1.00 |
| Kato et al. [4] | 5.78 | 2019 | 2020 | 2022 | 5 | 0.01 | 1.04 |
| Furlong [2] | 5.13 | 2008 | 2012 | 2016 | 4 | 0.04 | 1.20 |
| Tateno et al. [11] | 5.23 | 2012 | 2015 | 2019 | 4 | 0.01 | 1.08 |
| Teo and Gaw [14] | 5.22 | 2010 | 2013 | 2018 | 5 | 0.00 | 1.00 |
| Kondo et al. [8] | 4.67 | 2013 | 2016 | 2019 | 3 | 0.05 | 1.26 |
| Kato et al. [3] | 4.57 | 2018 | 2018 | 2020 | 2 | 0.03 | 1.17 |
| Kato et al. [5] | 4.41 | 2020 | 2020 | 2022 | 2 | 0.02 | 1.11 |
| Teo et al. [13] | 4.38 | 2015 | 2020 | 2022 | 2 | 0.03 | 1.15 |
| Yong and Nomura [15] | 4.07 | 2019 | 2020 | 2022 | 2 | 0.08 | 1.36 |
| Krieg and Dickie [9] | 3.79 | 2013 | 2016 | 2019 | 3 | 0.01 | 1.03 |
| Kato et al. [6] | 3.40 | 2012 | 2018 | 2020 | 2 | 0.00 | 1.00 |
| Chauliac et al. [1] | 3.38 | 2012 | 2018 | 2020 | 2 | 0.01 | 1.03 |
| Saito and Angles [10] | 3.14 | 2013 | 2017 | 2022 | 5 | 0.01 | 1.17 |

### *1.4. Country analysis*

The country analysis generated a network with 47 nodes (i.e., countries) and 97 links. An total of 5 countries showed a citation burst when $\gamma = 0.60$. The parameter $\gamma$ modulates the sensitivity of the node's burst detection [7]. The five countries with a citation burst were the United States, Switzerland, Hong Kong, France and Singapore (Table S3).

The main countries included in the network were mostly post-industrial societies, which is in line with the conceptualisation of *hikikomori* as a society-bound syndrome associated with the demands of a modern society.

**Table S3.** Five countries with a citation burst.

| Country | Strength | Burst Begin | Burst End | Duration |
|---|---|---|---|---|
| France | 3.33 | 2017 | 2018 | 1 |
| United States | 3.16 | 2007 | 2013 | 6 |
| Hong Kong | 2.49 | 2014 | 2018 | 4 |
| Switzerland | 1.93 | 2011 | 2013 | 2 |
| Singapore | 1.88 | 2018 | 2020 | 2 |

## References


[1] Chauliac, N., Couillet, A., Faivre, S., Brochard, N., and Terra, J.-L. (2017). Characteristics of socially withdrawn youth in france: a retrospective study. *International Journal of Social Psychiatry*, 63(4):339–344.

[2] Furlong, A. (2008). The japanese hikikomori phenomenon: acute social withdrawal among young people. *The sociological review*, 56(2):309–325.

[3] Kato, T. A., Kanba, S., and Teo, A. R. (2018). Hikikomori: experience in japan and international relevance. *World psychiatry*, 17(1):105.

[4] Kato, T. A., Kanba, S., and Teo, A. R. (2019). Hikikomori: multidimensional understanding, assessment, and future international perspectives. *Psychiatry and clinical neurosciences*, 73(8):427–440.

[5] Kato, T. A., Kanba, S., and Teo, A. R. (2020). Defining pathological social withdrawal: proposed diagnostic criteria for hikikomori. *World Psychiatry*, 19(1):116–117.

[6] Kato, T. A., Tateno, M., Shinfuku, N., Fujisawa, D., Teo, A. R., Sartorius, N., Akiyama, T., Ishida, T., Choi, T. Y., Balhara, Y. P. S., et al. (2012). Does the 'hikikomori'syndrome of social withdrawal exist outside japan? a preliminary international investigation. *Social psychiatry and psychiatric epidemiology*, 47(7):1061–1075.

[7] Kleinberg, J. (2003). Bursty and hierarchical structure in streams. *Data mining and knowledge discovery*, 7(4):373–397.

[8] Kondo, N., Sakai, M., Kuroda, Y., Kiyota, Y., Kitabata, Y., and Kurosawa, M. (2013). General condition of hikikomori (prolonged social withdrawal) in japan: psychiatric diagnosis and outcome in mental health welfare centres. *International Journal of Social Psychiatry*, 59(1):79–86.

[9] Krieg, A. and Dickie, J. R. (2013). Attachment and hikikomori: A psychosocial developmental model. *International Journal of Social Psychiatry*, 59(1):61–72.

[10] Saito, T. and Angles, J. T. (2013). *Hikikomori: Adolescence without end.* University of Minnesota Press.

[11] Tateno, M., Park, T. W., Kato, T. A., Umene-Nakano, W., and Saito, T. (2012). Hikikomori as a possible clinical term in psychiatry: a questionnaire survey. *BMC psychiatry*, 12(1):1–7.

[12] Teo, A. R. (2010). A new form of social withdrawal in japan: a review of hikikomori. *International journal of social psychiatry*, 56(2):178–185.

[13] Teo, A. R., Fetters, M. D., Stufflebam, K., Tateno, M., Balhara, Y., Choi, T. Y., Kanba, S., Mathews, C. A., and Kato, T. A. (2015). Identification of the hikikomori syndrome of social withdrawal: psychosocial features and treatment preferences in four countries. *International Journal of Social Psychiatry*, 61(1):64–72.

[14] Teo, A. R. and Gaw, A. C. (2010). Hikikomori, a japanese culture-bound syndrome of social withdrawal? a proposal for dsm-v. *The Journal of nervous and mental disease*, 198(6):444.

[15] Yong, R. and Nomura, K. (2019). Hikikomori is most associated with interpersonal relationships, followed by suicide risks: a secondary analysis of a national cross-sectional study. *Frontiers in psychiatry*, 10:247.

## 2. Cited References in each cluster

The list of cited references in each cluster are summarised in the following table.

| Freq | Author | Year | TITLE | ClusterID |
|---|---|---|---|---|
| 12 | Teo AR | 2018 | DEVELOPMENT AND VALIDATION OF THE 25-ITEM HIKIKOMORI QUESTIONNAIRE (HQ-25) @ PSYCHIATRY CLIN NEUROSCI | 0 |
| 14 | Kato TA | 2018 | HIKIKOMORI | 0 |
| 10 | Malag√≥n-Amor √Å | 2018 | A 12-MONTH STUDY OF THE HIKIKOMORI SYNDROME OF SOCIAL WITHDRAWAL | 0 |
| 11 | Teo AR | 2015 | PSYCHOPATHOLOGY ASSOCIATED WITH SOCIAL WITHDRAWAL | 0 |
| 11 | Stip E | 2016 | INTERNET ADDICTION, HIKIKOMORI SYNDROME, AND THE PRODROMAL PHASE OF PSYCHOSIS @ FRONT PSYCHIATRY | 0 |
| 26 | Teo AR | 2015 | IDENTIFICATION OF THE HIKIKOMORI SYNDROME OF SOCIAL WITHDRAWAL | 0 |
| 10 | Chauliac N | 2017 | CHARACTERISTICS OF SOCIALLY WITHDRAWN YOUTH IN FRANCE | 0 |
| 4 | Kato TA | 2011 | ARE JAPANS HIKIKOMORI AND DEPRESSION IN YOUNG PEOPLE SPREADING ABROAD? | 0 |
| 28 | Kondo N | 2013 | GENERAL CONDITION OF HIKIKOMORI (PROLONGED SOCIAL WITHDRAWAL) IN JAPAN | 0 |
| 7 | Ovejero S | 2014 | PROLONGED SOCIAL WITHDRAWAL DISORDER | 0 |
| 17 | Kato TA | 2019 | HIKIKOMORI | 0 |
| 12 | Kato TA | 2012 | DOES THE HIKIKOMORI SYNDROME OF SOCIAL WITHDRAWAL EXIST OUTSIDE JAPAN? | 0 |
| 14 | Lee YS | 2013 | HOME VISITATION PROGRAM FOR DETECTING, EVALUATING AND TREATING SOCIALLY WITHDRAWN YOUTH IN KOREA @ PSYCHIATRY CLIN NEUROSCI | 0 |
| 4 | Kato TA | 2016 | MULTIDIMENSIONAL ANATOMY OF MODERN TYPE DEPRESSION IN JAPAN | 0 |
| 3 | Kato TA | 2017 | LONELINESS AND SINGLE-PERSON HOUSEHOLDS | 0 |
| 10 | Wong PW | 2015 | THE PREVALENCE AND CORRELATES OF SEVERE SOCIAL WITHDRAWAL (HIKIKOMORI) IN HONG KONG | 0 |
| 2 | Kato TA | 2018 | IS A SOCIO-CULTURAL ANALYSIS OF DEPRESSIVE DISORDERS A MATTER OF CONCERN? | 0 |
| 2 | Kato TA | 2017 | MODERN-TYPE DEPRESSION AS AN "ADJUSTMENT" DISORDER IN JAPAN | 0 |

| | | | | |
|---|---|---|---|---|
| 2 | Kashihara J | 2019 | PERCEPTIONS OF TRADITIONAL AND MODERN TYPES OF DEPRESSION | 0 |
| 8 | Wong PWC | 2017 | DOES HIKIKOMORI (SEVERE SOCIAL WITHDRAWAL) EXIST AMONG YOUNG PEOPLE IN URBAN AREAS OF CHINA? | 0 |
| 2 | Kuwano N | 2018 | TRYPTOPHAN-KYNURENINE AND LIPID RELATED METABOLITES AS BLOOD BIOMARKERS FOR FIRST-EPISODE DRUG-NAIVE PATIENTS WITH MAJOR DEPRESSIVE DISORDER | 0 |
| 2 | Kato TA | 2016 | BOUNDLESS SYNDROMES IN MODERN SOCIETY | 0 |
| 2 | Kato TA | 2013 | NEURON-GLIA INTERACTION AS A POSSIBLE GLUE TO TRANSLATE THE MIND-BRAIN GAP | 0 |
| 2 | Pontes HM | 2017 | THE DEVELOPMENT AND PSYCHOMETRIC EVALUATION OF THE INTERNET DISORDER SCALE (IDS-15) @ ADDICT BEHAV | 0 |
| 2 | Monacis L | 2018 | ASSESSMENT OF THE ITALIAN VERSION OF THE INTERNET DISORDER SCALE (IDS-15) @ INT J MENT HEALTH ADDICT | 0 |
| 3 | Amendola S | 2021 | HIKIKOMORI, PROBLEMATIC INTERNET USE AND PSYCHOPATHOLOGY | 0 |
| 2 | Ferrara P | 2020 | THE HIKIKOMORI PHENOMENON OF SOCIAL WITHDRAWAL | 0 |
| 2 | Tajan N | 2017 | HIKIKOMORI | 0 |
| 2 | Ranieri F | 2018 | HIKIKOMORI | 0 |
| 2 | Fossati A | 2017 | THE PERSONALITY INVENTORY FOR DSM-5 BRIEF FORM | 0 |
| 8 | Nagata T | 2013 | COMORBID SOCIAL WITHDRAWAL (HIKIKOMORI) IN OUTPATIENTS WITH SOCIAL ANXIETY DISORDER | 0 |
| 11 | Gondim FAA | 2017 | HIKIKOMORI IN BRAZIL | 0 |
| 1 | Bogousslavsky J | 2011 | HYSTERIA AFTER CHARCOT | 0 |
| 2 | Setoyama D | 2016 | PLASMA METABOLITES PREDICT SEVERITY OF DEPRESSION AND SUICIDAL IDEATION IN PSYCHIATRIC PATIENTS | 0 |
| 2 | Malagon-Amor A | 2018 | A 12-MONTH STUDY OF THE HIKIKOMORI SYNDROME OF SOCIAL WITHDRAWAL | 0 |

| | | | | |
|---|---|---|---|---|
| 1 | Avcil S | 2018 | EVALUATION OF THE NEUTROPHIL/LYMPHOCYTE RATIO, PLATELET/LYMPHOCYTE RATIO, AND MEAN PLATELET VOLUME AS INFLAMMATORY MARKERS IN CHILDREN WITH ATTENTION-DEFICIT HYPERACTIVITY DISORDER @ PSYCHIATRY CLIN NEUROSCI | 0 |
| 4 | Kato TA | 2016 | A 39-YEAR-OLD ‚ÄúADULTOLESCENT | 0 |
| 13 | Kato TA | 2020 | DEFINING PATHOLOGICAL SOCIAL WITHDRAWAL | 0 |
| 1 | Bloomfield PS | 2016 | MICROGLIAL ACTIVITY IN PEOPLE AT ULTRA HIGH RISK OF PSYCHOSIS AND IN SCHIZOPHRENIA | 0 |
| 1 | Amendola S | 2021 | PATTERNS OF INTERNALIZING SYMPTOMS AND DISABILITY FUNCTIONING IN CHILDREN AND ADOLESCENTS | 0 |
| 1 | Angane A | 2021 | PSYCHOSIS UNMASKED BY GAMING | 0 |
| 1 | Amankwaa EF | 2018 | CITIES AT RISK? | 0 |
| 1 | Andrie EK | 2019 | GAMBLING INVOLVEMENT AND PROBLEM GAMBLING CORRELATES AMONG EUROPEAN ADOLESCENTS | 0 |
| 18 | Teo AR | 2010 | A NEW FORM OF SOCIAL WITHDRAWAL IN JAPAN | 0 |
| 1 | Adawi M | 2019 | PSYCHOMETRIC PROPERTIES OF THE BRIEF SYMPTOM INVENTORY IN NOMOPHOBIC SUBJECTS | 0 |
| 1 | Brand M | 2014 | INTERNET ADDICTION | 0 |
| 1 | Amendola S | 2022 | PSYCHOMETRIC PROPERTIES OF THE ITALIAN VERSION OF THE 25-ITEM HIKIKOMORI QUESTIONNAIRE | 0 |
| 7 | Krieg A | 2013 | ATTACHMENT AND HIKIKOMORI | 0 |
| 4 | Wu AFW | 2019 | EVIDENCE OF PATHOLOGICAL SOCIAL WITHDRAWAL IN NON-ASIAN COUNTRIES | 0 |
| 2 | Kato TA | 2017 | CAN POKEMON GO RESCUE SHUT-INS (HIKIKOMORI) FROM THEIR ISOLATED WORLD? | 0 |
| 4 | Hamasaki Y | 2021 | IDENTIFYING SOCIAL WITHDRAWAL (HIKIKOMORI) FACTORS IN ADOLESCENTS | 0 |

| | | | | |
|---|---|---|---|---|
| 9 | Malag√≥n-Amor √Å | 2015 | HIKIKOMORI IN SPAIN | 0 |
| 13 | Li TM | 2015 | YOUTH SOCIAL WITHDRAWAL BEHAVIOR (HIKIKOMORI) | 0 |
| 7 | Katsuki R | 2019 | CLARIFYING DEEPER PSYCHOLOGICAL CHARACTERISTICS OF HIKIKOMORI USING THE RORSCHACH COMPREHENSIVE SYSTEM | 0 |
| 3 | Martinotti G | 2021 | HIKIKOMORI | 0 |
| 4 | Bowker JC | 2019 | SEVERE SOCIAL WITHDRAWAL | 0 |
| 2 | Bommersbach T | 2019 | NO LONGER CULTURE-BOUND | 0 |
| 4 | Koyama A | 2010 | LIFETIME PREVALENCE, PSYCHIATRIC COMORBIDITY AND DEMOGRAPHIC CORRELATES OF ,ÄúHIKIKOMORI,Äù IN A COMMUNITY POPULATION IN JAPAN @ PSYCHIATRY RES | 0 |
| 6 | Frankova I | 2019 | SIMILAR BUT DIFFERENT | 0 |
| 4 | Yamamoto A | 2014 | CRAFT APPLICATION IN AN INTERVENTION PROGRAM FOR HIKIKOMORI CASES WITH (SUSPECTED) AUTISM SPECTRUM DISORDER | 0 |
| 8 | Kato TA | 2012 | DOES THE ,ÄòHIKIKOMORI,Äô SYNDROME OF SOCIAL WITHDRAWAL EXIST OUTSIDE JAPAN? | 0 |
| 2 | Yasuma N | 2021 | PSYCHOTIC EXPERIENCES AND HIKIKOMORI IN A NATIONALLY REPRESENTATIVE SAMPLE OF ADULT COMMUNITY RESIDENTS IN JAPAN | 0 |
| 9 | Teo AR | 2013 | SOCIAL ISOLATION ASSOCIATED WITH DEPRESSION | 0 |
| 1 | Abe T | 2017 | SHIN-GATA UTSU TO HATTATSU SHOUGAI NEW TYPE DEPRESSION AND DEVELOPMENTAL DISORDERS @ JAPANESE JOURNAL OF PSYCHIATRY | 0 |
| 2 | Pozza A | 2019 | THE ,ÄòHIKIKOMORI,Äô SYNDROME | 0 |
| 1 | 5TH EDN | 2013 | DIAGNOSTIC AND STATISTICAL MANUAL OF MENTAL DISORDERS | 0 |
| 2 | Wong PW | 2014 | THE PREVALENCE AND CORRELATES OF SEVERE SOCIAL WITHDRAWAL (HIKIKOMORI) IN HONG KONG | 0 |
| 2 | Aguglia E | 2010 | HIKIKOMORI PHENOMENON | 0 |
| 2 | Wong JCM | 2019 | HIKIKOMORI PHENOMENON IN EAST ASIA | 0 |

| | | | | |
|---|---|---|---|---|
| 1 | Amendola S | 2022 | PSYCHOMETRIC PROPERTIES OF THE ITALIAN VERSION OF THE 25-ITEM HIKIKOMORI QUESTIONNAIRE FOR ADOLESCENTS | 0 |
| 3 | Liu LL | 2018 | HARNESSING SOCIAL MEDIA TO EXPLORE YOUTH SOCIAL WITHDRAWAL IN THREE MAJOR CITIES IN CHINA | 0 |
| 14 | Teo AR | 2010 | HIKIKOMORI, A JAPANESE CULTURE-BOUND SYNDROME OF SOCIAL WITHDRAWAL? | 0 |
| 2 | Yong R | 2020 | CHARACTERISTICS OF AND GENDER DIFFERENCE FACTORS OF HIKIKOMORI AMONG THE WORKING-AGE POPULATION | 0 |
| 6 | Teo AR | 2020 | SOCIAL WITHDRAWAL IN MAJOR DEPRESSIVE DISORDER | 0 |
| 2 | Nonaka S | 2018 | ASSESSING ADAPTIVE BEHAVIORS OF INDIVIDUALS WITH HIKIKOMORI (PROLONGED SOCIAL WITHDRAWAL) | 0 |
| 3 | Liu LL | 2018 | HARNESSING SOCIAL MEDIA TO EXPLORE YOUTH SOCIAL WITHDRAWAL IN THREE MAJOR CITIES IN CHINA | 0 |
| 3 | Nonaka S | 2020 | FAMILY BEHAVIORAL REPERTOIRES AND FAMILY INTERACTION INFLUENCE THE ADAPTIVE BEHAVIORS OF INDIVIDUALS WITH HIKIKOMORI @ FRONTIERS IN PSYCHIATRY | 0 |
| 5 | Wong PWC | 2015 | THE PREVALENCE AND CORRELATES OF SEVERE SOCIAL WITHDRAWAL (HIKIKOMORI) IN HONG KONG | 0 |
| 2 | Tateno M | 2019 | INTERNET ADDICTION, SMARTPHONE ADDICTION, AND HIKIKOMORI TRAIT IN JAPANESE YOUNG ADULT | 0 |
| 2 | Nonaka S | 2021 | A CORRELATIONAL STUDY OF SOCIOECONOMIC FACTORS AND THE PREVALENCE OF HIKIKOMORI IN JAPAN FROM 2010 TO 2019 @ COMPREHENSIVE PSYCHIATRY | 0 |
| 3 | Uchida Y | 2015 | THE NEET AND HIKIKOMORI SPECTRUM | 0 |

| | | | | |
|---|---|---|---|---|
| 1 | Gender Equality Bureau, Cabinet Office, Government of Japan | 2020 | Women and men in Japan; 2020:12. Pamphlet available at https://www.gender.go.jp/english_contents/pr_act/pub/pamphlet/women-and-men20/index.html | 0 |
| 2 | Hayakawa K | 2018 | BLOOD BIOMARKERS OF HIKIKOMORI, A SEVERE SOCIAL WITHDRAWAL SYNDROME @ SCI REP | 0 |
| 2 | Sakai M | 2015 | EFFECTIVENESS OF COMMUNITY REINFORCEMENT AND FAMILY TRAINING (CRAFT) FOR PARENTS OF INDIVIDUALS WITH ,ÄòHIKIKOMORI,Äô @ JAPANESE JOURNAL OF BEHAVIOR THERAPY | 0 |
| 1 | Arroll B | 2010 | VALIDATION OF PHQ-2 AND PHQ-9 TO SCREEN FOR MAJOR DEPRESSION IN THE PRIMARY CARE POPULATION @ ANN FAM MED | 0 |
| 1 | Campbell LE | 2018 | SEVERITY OF ILLNESS AND ADAPTIVE FUNCTIONING PREDICT QUALITY OF CARE OF CHILDREN AMONG PARENTS WITH PSYCHOSIS | 0 |
| 1 | Barnes AS | 2012 | OBESITY AND SEDENTARY LIFESTYLES | 0 |
| 1 | Arima H | 2012 | EFFECTS OF PREHYPERTENSION AND HYPERTENSION SUBTYPE ON CARDIOVASCULAR DISEASE IN THE ASIA-PACIFIC REGION @ HYPERTENSION | 0 |
| 1 | Almeida JBSAD | 2017 | POPULA√á√ÉO NEM-NEM | 0 |
| 1 | Campbell L | 2012 | THE EXPERIENCES OF AUSTRALIAN PARENTS WITH PSYCHOSIS | 0 |
| 2 | Malag√≥n-Amor √Å | 2020 | FAMILY FEATURES OF SOCIAL WITHDRAWAL SYNDROME (HIKIKOMORI) @ FRONTIERS IN PSYCHIATRY | 0 |
| 1 | 5TH ED | 2013 | DIAGNOSTIC AND STATISTICAL MANUAL OF MENTAL DISORDERS. | 0 |
| 2 | Funakoshi A | 2015 | SIGNIFICANT FACTORS IN FAMILY DIFFICULTIES FOR FATHERS AND MOTHERS WHO USE SUPPORT SERVICES FOR CHILDREN WITH HIKIKOMORI @ PSYCHIATRY AND CLINICAL NEUROSCIENCES | 0 |

| | | | | |
|---|---|---|---|---|
| 1 | Barbaresi WJ | 2013 | MORTALITY, ADHD, AND PSYCHOSOCIAL ADVERSITY IN ADULTS WITH CHILDHOOD ADHD | 0 |
| 1 | Allison A | 2006 | @ MILLENNIAL JAPAN INTIMATE ALIENATION AND NEW AGE INTIMACIES | 0 |
| 1 | Brooks SK | 2020 | THE PSYCHOLOGICAL IMPACT OF QUARANTINE AND HOW TO REDUCE IT | 0 |
| 3 | Kubo H | 2020 | DEVELOPMENT OF 5-DAY HIKIKOMORI INTERVENTION PROGRAM FOR FAMILY MEMBERS | 0 |
| 12 | Yong R | 2019 | HIKIKOMORI IS MOST ASSOCIATED WITH INTERPERSONAL RELATIONSHIPS, FOLLOWED BY SUICIDE RISKS | 1 |
| 11 | Malag√≥n-Amor √Å | 2015 | HIKIKOMORI IN SPAIN | 1 |
| 16 | Kondo N | 2013 | GENERAL CONDITION OF HIKIKOMORI (PROLONGED SOCIAL WITHDRAWAL) IN JAPAN | 1 |
| 15 | Ovejero S | 2014 | PROLONGED SOCIAL WITHDRAWAL DISORDER | 1 |
| 8 | Tajan N | 2015 | SOCIAL WITHDRAWAL AND PSYCHIATRY | 1 |
| 16 | Teo AR | 2015 | IDENTIFICATION OF THE HIKIKOMORI SYNDROME OF SOCIAL WITHDRAWAL | 1 |
| 1 | Ballarotto G | 2018 | ADOLESCENT INTERNET ABUSE | 1 |
| 7 | Kato TA | 2020 | DEFINING PATHOLOGICAL SOCIAL WITHDRAWAL | 1 |
| 8 | Kato TA | 2019 | HIKIKOMORI | 1 |
| 1 | Antai-Otong D | 2016 | PSYCHOSOCIAL RECOVERY AND REHABILITATION @ NURS CLIN N AM | 1 |
| 5 | Ranieri F | 2015 | WHEN SOCIAL WITHDRAWAL IN ADOLESCENCE BECOMES EXTREME | 1 |
| 2 | Kato TA | 2017 | CAN POK√âMON GO RESCUE SHUT-INS (HIKIKOMORI) FROM THEIR ISOLATED WORLD? | 1 |
| 5 | Lee YS | 2013 | HOME VISITATION PROGRAM FOR DETECTING, EVALUATING AND TREATING SOCIALLY WITHDRAWN YOUTH IN KOREA @ PSYCHIATRY CLIN NEUROSCI | 1 |
| 6 | Teo AR | 2010 | HIKIKOMORI, A JAPANESE CULTURE-BOUND SYNDROME OF SOCIAL WITHDRAWAL? | 1 |

| | | | | |
|---|---|---|---|---|
| 9 | Krieg A | 2013 | ATTACHMENT AND HIKIKOMORI | 1 |
| 7 | Tateno M | 2019 | INTERNET ADDICTION, SMARTPHONE ADDICTION, AND HIKIKOMORI TRAIT IN JAPANESE YOUNG ADULT | 1 |
| 3 | Chauliac N | 2017 | CHARACTERISTICS OF SOCIALLY WITHDRAWN YOUTH IN FRANCE | 1 |
| 2 | Kato TA | 2017 | MODERN-TYPE DEPRESSION AS AN ‚ÄúADJUSTMENT | 1 |
| 2 | Kato TA | 2019 | DEVELOPMENT AND VALIDATION OF THE 22-ITEM TARUMI‚ÄôS MODERN-TYPE DEPRESSION TRAIT SCALE | 1 |
| 2 | Kubo H | 2020 | DEVELOPMENT OF 5-DAY HIKIKOMORI INTERVENTION PROGRAM FOR FAMILY MEMBERS | 1 |
| 2 | Kubo H | 2021 | DEVELOPMENT OF A 3-DAY INTERVENTION PROGRAM FOR FAMILY MEMBERS OF HIKIKOMORI SUFFERERS @ JAPANESE PSYCHOLOGICAL RESEARCH | 1 |
| 2 | Kato TA | 2016 | MULTIDIMENSIONAL ANATOMY OF ‚ÄòMODERN TYPE DEPRESSION‚Äô IN JAPAN | 1 |
| 2 | Rubinstein E | 2016 | EMPLOTTING HIKIKOMORI | 1 |
| 3 | Teo AR | 2018 | DEVELOPMENT AND VALIDATION OF THE 25-ITEM HIKIKOMORI QUESTIONNAIRE (HQ-25) @ PSYCHIATRY AND CLINICAL NEUROSCIENCES | 1 |
| 2 | 5 | 2013 | DIAGNOSTIC AND STATISTICAL MANUAL OF MENTAL DISORDERS | 1 |
| 2 | Aguglia E | 2010 | IL FENOMENO DELLHIKIKOMORI | 1 |
| 1 | Carratelli TI | 2015 | ‚Äò√à TUTTA UNA QUESTIONE DI SGUARDI E DI NASCONDIGLI‚Äô NOTE TEORICO-CLINICHE SULLADOLESCENTE RITIRATO IN S√â @ PSICOANALISI | 1 |
| 1 | Bowker MH | 2016 | JOURNAL OF PSYCHO-SOCIAL STUDIES | 1 |
| 1 | Argese MG | 2011 | @ REPORT DI MARIA GIOVANNA ARGESE DEL CONVEGNO ‚ÄòRIFUGI DELLA MENTE‚Äô 1‚Äì2 OTTOBRE 2011 | 1 |
| 1 | Bale TL | 2015 | SEX DIFFERENCES AND STRESS ACROSS THE LIFESPAN @ NATURE NEUROSCIENCE | 1 |
| 1 | Arriola-Vigo JA | 2019 | PERCEPTIONS OF COMMUNITY INVOLVEMENT IN THE PERUVIAN MENTAL HEALTH REFORM PROCESS AMONG CLINICIANS AND POLICY-MAKERS | 1 |

| | | | | |
|---|---|---|---|---|
| 1 | Bayless DW | 2016 | PHILOSOPHICAL TRANSACTIONS OF THE ROYAL SOCIETY B | 1 |
| 4 | Todd KHL | 2011 | HIKIKOMANIA | 1 |
| 1 | Antezana L | 2019 | GENDER DIFFERENCES IN RESTRICTED AND REPETITIVE BEHAVIORS AND INTERESTS IN YOUTH WITH AUTISM @ AUTISM RESEARCH | 1 |
| 5 | Uchida Y | 2015 | THE NEET AND HIKIKOMORI SPECTRUM | 1 |
| 1 | Barlati S | 2020 | SOCIAL COGNITION IN A RESEARCH DOMAIN CRITERIA PERSPECTIVE | 1 |
| 1 | Akbari M | 2021 | CLINICAL PSYCHOLOGY & PSYCHOTHERAPY | 1 |
| 2 | Imai H | 2020 | THE CHARACTERISTICS AND SOCIAL FUNCTIONING OF PATHOLOGICAL SOCIAL WITHDRAWAL, ‚ÄúHIKIKOMORI, | 1 |
| 2 | Stip E | 2016 | INTERNET ADDICTION, HIKIKOMORI SYNDROME, AND THE PRODROMAL PHASE OF PSYCHOSIS @ FRONTIERS IN PSYCHIATRY | 1 |
| 4 | Norasakkun kit V | 2014 | TO CONFORM OR TO MAINTAIN SELF-CONSISTENCY? | 1 |
| 1 | Beggiato A | 2016 | GENDER DIFFERENCES IN AUTISM SPECTRUM DISORDERS | 1 |
| 2 | Wong J | 2019 | HIKIKOMORI PHENOMENON IN EAST ASIA | 1 |
| 2 | Bowker JC | 2019 | SEVERE SOCIAL WITHDRAWAL | 1 |
| 6 | Kato TA | 2020 | INTERNET SOCIETY, INTERNET ADDICTION, AND PATHOLOGICAL SOCIAL WITHDRAWAL | 1 |
| 2 | Kato TA | 2019 | DEVELOPMENT AND VALIDATION OF THE 22-ITEM TARUMIS MODERN-TYPE DEPRESSION TRAIT SCALE | 1 |
| 3 | Haasio A | 2019 | INFORMATION NEEDS OF THE FINNISH AND JAPANESE HIKIKOMORI | 1 |
| 4 | Wong V | 2009 | YOUTH LOCKED IN TIME AND SPACE? | 1 |
| 2 | Malag√≥n-Amor √Å | 2020 | FAMILY FEATURES OF SOCIAL WITHDRAWAL SYNDROME (HIKIKOMORI) @ FRONTIERS IN PSYCHIATRY | 1 |
| 2 | Nagata T | 2013 | COMORBID SOCIAL WITHDRAWAL (HIKIKOMORI) IN OUTPATIENTS WITH SOCIAL ANXIETY DISORDER | 1 |
| 2 | Tan M | 2020 | INTERNATIONAL EXPERIENCE OF HIKIKOMORI (PROLONGED SOCIAL WITHDRAWAL) AND ITS RELEVANCE TO PSYCHIATRIC RESEARCH | 1 |
| 5 | Teo AR | 2013 | SOCIAL ISOLATION ASSOCIATED WITH DEPRESSION | 1 |

| | | | | |
|---|---|---|---|---|
| 3 | Teo AR | 2020 | SOCIAL WITHDRAWAL IN MAJOR DEPRESSIVE DISORDER | 1 |
| 1 | Arnett J | 2015 | @ EMERGING ADULTHOOD | 1 |
| 1 | Backhaus J | 2013 | @ HIKIKOMORI AND THE RENTAL SISTER | 1 |
| 1 | Cacioppo JT | 2015 | THE NEUROENDOCRINOLOGY OF SOCIAL ISOLATION @ ANNU REV PSYCHOL | 1 |
| 1 | Backhaus J | 2013 | @ HIKIKOMORI | 1 |
| 1 | Addati L | 2018 | CARE WORK AND CARE JOBS FOR THE FUTURE OF DECENT WORK | 1 |
| 1 | Arai H | 2015 | SYAKOU-HUAN SHYOUJOU TO TAIJINN-TEKI JIKO-KOURYOKUKAN GA DAIGAKUSEI NO HIKIKOMORI-SHINWASEI NI ATAERU EIKYOU THE EFFECTS OF SOCIAL ANXIETY SYMPTOMS AND SOCIAL SELF-EFFICACY ON AFFINITY FOR SOCIAL WITHDRAWAL IN UNIVERSITY STUDENTS @ JAPANESE JOURNAL OF PERSONALITY | 1 |
| 2 | Frankova I | 2019 | SIMILAR BUT DIFFERENT | 1 |
| 1 | Abrams LS | 2010 | SAMPLING ‚ÄòHARD TO REACH‚Äô POPULATIONS IN QUALITATIVE RESEARCH | 1 |
| 8 | Li TM | 2015 | YOUTH SOCIAL WITHDRAWAL BEHAVIOR (HIKIKOMORI) | 1 |
| 2 | Heinze U | 2014 | SELF AND SALVATION | 1 |
| 1 | Bor W | 2014 | ARE CHILD AND ADOLESCENT MENTAL HEALTH PROBLEMS INCREASING IN THE 21ST CENTURY? | 1 |
| 1 | Benke C | 2020 | LOCKDOWN, QUARANTINE MEASURES, AND SOCIAL DISTANCING | 1 |
| 1 | 2019 2019 Retrieved from | 2019 | 2019 2019 RETRIEVED FROM | 1 |
| 2 | Katsuki R | 2020 | AUTISM SPECTRUM CONDITIONS IN HIKIKOMORI | 1 |
| 2 | Tateno M | 2012 | HIKIKOMORI AS A POSSIBLE CLINICAL TERM IN PSYCHIATRY | 1 |
| 9 | Teo AR | 2010 | A NEW FORM OF SOCIAL WITHDRAWAL IN JAPAN | 1 |
| 1 | Armstrong M | 2016 | 2019) @ @ | 1 |
| 5 | Suwa M | 2013 | THE PHENOMENON OF ‚ÄúHIKIKOMORI | 1 |

| | | | | |
|---|---|---|---|---|
| 1 | Garcv≠a-Campayo J | 2007 | A CASE REPORT OF HIKIKOMORI IN SPAIN @ MED CLIN | 1 |
| 5 | Borovoy A | 2008 | JAPANS HIDDEN YOUTHS | 3 |
| 1 | Allison A | 2009 | THE AFFECTIVE ACTIVISM OF JAPANESE YOUTH @ THEORY | 3 |
| 1 | Adachi N | 2006 | @ OYA TO HANARETE | 3 |
| 1 | Brinton M | 2011 | CAMBRIDGE UNIVERSITY PRESS @ @ LOST IN TRANSITION | 3 |
| 1 | Asai J | 2005 | @ Nƒ™TO TO IWARERU HITOBITO | 3 |
| 12 | Furlong A | 2008 | THE JAPANESE HIKIKOMORI PHENOMENON | 3 |
| 1 | ASAHI SHIMBUN | 2005 | WAKAMONO NO JIRITSU: SAISHO NO IPPO WO SASAEY≈å | 3 |
| 2 | De Michele F | 2013 | HIKIKOMORI | 3 |
| 3 | Garcia-Campayo J | 2007 | A CASE REPORT OF HIKIKOMORI IN SPAIN @ MED CLIN (BARC) | 3 |
| 1 | Block JJ | 2008 | ISSUES FOR DSM-V | 3 |
| 1 | Bartels M | 2004 | GENETIC AND ENVIRONMENTAL MECHANISMS UNDERLYING STABILITY AND CHANGE IN PROBLEM BEHAVIORS AT AGES 3, 7, 10, AND 12 @ DEVELOPMENTAL PSYCHOLOGY | 3 |
| 1 | Kondo N | 2008 | SHISHUNKI HIKIKOMORI NI OKERU SEISHIN IGAKUTEKI SHOUGAI NO JITTAI HAAKU NI KAN SURU KENKYUU | 3 |
| 1 | Jorm AF | 2005 | PUBLIC BELIEFS ABOUT TREATMENT AND OUTCOME OF MENTAL DISORDERS | 3 |
| 1 | Degnan KA | 2007 | PII S0954579407000363 @ BEHAVIORAL INHIBITION AND ANXIETY DISORDERS | 3 |
| 1 | Cooper PJ | 2006 | PII S016503270600142X @ AFFECTIVE DISORDER IN THE PARENTS OF A CLINIC SAMPLE OF CHILDREN WITH ANXIETY DISORDERS @ JOURNAL OF AFFECTIVE DISORDERS | 3 |
| 1 | Kiyota A | 2008 | CHIIKI RENKEI SHISUTEMU NI YORU HIKIKOMORI SHIEN TO EKIGAKUTEKI KENTOU | 3 |

| | | | | |
|---|---|---|---|---|
| 1 | Genda Y | 2007 | JOBLESS YOUTHS AND THE NEET PROBLEM IN JAPAN @ SOCIAL SCIENCE JAPAN JOURNAL | 3 |
| 1 | Pies R | 2009 | SHOULD DSM-V DESIGNATE "INTERNET ADDICTION" A MENTAL DISORDER? | 3 |
| 1 | Aguglia E | 2010 | HIKIKOMORI PHENOMENON | 3 |
| 3 | Jones M | 2006 | SHUTTING THEMSELVES IN @ NEW YORK TIMES MAGAZINE | 3 |
| 1 | Honda Y | 2006 | @ Nƒ™TOTTE IU NA! | 3 |
| 1 | MINISTRY OF HEALTH | 2003 | 10-DAI | 3 |
| 1 | Gariup M | 2008 | HIKIKOMORI OR SIMPLE SCHIZOPHRENIA? | 3 |
| 1 | Genda Y | 2004 | 14-SAI NI "II OTONA" TO DEAWASEY≈åU @ CH≈™≈å K≈å≈™RON | 3 |
| 1 | KIKUZO II VISUAL FOR LIBRARY | 2009 | ASAHI SHIMBUN 2002-2008 | 3 |
| 1 | Genda Y | 2004 | @ NEET | 3 |
| 1 | Futagami N | 2005 | KIBO NO NITO | 3 |
| 1 | Horiguchi S | 2011 | HIKIKOMORI | 3 |
| 1 | Nakajima T | 2008 | SEISHINKA KYUUSEIKI IRYOU NI OKERU HIKIKOMORI SEINEN NO JITTAI TO SEISHIN IGAKUTEKI CHIRYOU NI KAN SURU KENKYUU | 3 |
| 1 | Hertog E | 2008 | THE WORST ABUSE AGAINST A CHILD IS THE ABSENCE OF A PARENT | 3 |
| 1 | Arnett J | 2014 | THE NEW LIFE STAGE OF EMERGING ADULTHOOD AT AGES 18-29 YEARS | 3 |
| 1 | Hattori Y | 2006 | SOCIAL WITHDRAWAL IN JAPANESE YOUTH @ J TRAUMA PRACT | 3 |
| 1 | Hughes AA | 2009 | ANXIETY, MOOD, AND SUBSTANCE USE DISORDERS IN PARENTS OF CHILDREN WITH ANXIETY DISORDERS @ CHILD PSYCHIATRY HUM DEV | 3 |
| 1 | Furuhashi T | 2011 | COMMONALITIES AND DIFFERENCES IN HIKIKOMORI YOUTHS IN JAPAN AND FRANCE @ NAGOYA JOURNAL OF HEALTH | 3 |
| 1 | Borovoy A | 2005 | @ THE TOO-GOOD WIFE | 3 |
| 1 | Hettema JM | 2005 | THE STRUCTURE OF GENETIC AND ENVIRONMENTAL RISK FACTORS FOR ANXIETY DISORDERS IN MEN AND WOMEN @ ARCHIVES OF GENERAL PSYCHIATRY | 3 |

| | | | | |
|---|---|---|---|---|
| 1 | Haro JM | 2006 | CONCORDANCE OF THE COMPOSITE INTERNATIONAL DIAGNOSTIC INTERVIEW VERSION 3 | 3 |
| 1 | Dickens AP | 2011 | INTERVENTIONS TARGETING SOCIAL ISOLATION IN OLDER PEOPLE | 3 |
| 1 | Fujita H | 2005 | HIKIKOMORI NO SHAKAITEKI-HAIKEI SOCIAL BACKGROUNDS OF HIKIKOMORI @ HIKIKOMORU WAKAMONO-TACHI WITHDRAWING YOUTH | 3 |
| 1 | Ide S | 2007 | @ HIKIKOMORI NO SHAKAIGAKU SOCIOLOGY OF HIKIKOMORI | 3 |
| 6 | Sakamoto N | 2005 | HIKIKOMORI, IS IT A CULTURE-REACTIVE OR CULTURE-BOUND SYNDROME? | 3 |
| 1 | Cardenal V | 2007 | @ ADAPTACI√ìN Y BAREMACI√ìN AL ESPA√ëOL DEL INVENTARIO CL√çNICO MULTIAXIAL DE MILLON-III (MCMI-III) | 3 |
| 1 | Cooperativa Minotauro La bruttezza immaginaria - Intervento clinico con i ragazzi ritirati Atti del | 2014 | COOPERATIVA MINOTAURO LA BRUTTEZZA IMMAGINARIA - INTERVENTO CLINICO CON I RAGAZZI RITIRATI ATTI DELLEVENTO CULTURALE TENUTOSI A MILANO IL 9 E 10 MAGGIO 2014 HTTP://WWWTELECOMITALIACOM/CONTENT/DAM/TELECOMITALIA/GENERALISTIC-IMAGES/DOCUMENTI/RICERCAPDF | 3 |
| 1 | Ciufferi MG | 2011 | MILANO - UDINE @ ESPERIENZE TERAPEUTICHE CON ADOLESCENTI CHE SI ISOLANO @ HIKIKOMORI E ADOLESCENZA - FENOMENOLOGIA DELLAUTORECLUSIONE | 3 |
| 1 | Gariup M | 2008 | HIKIKOMORI O ESQUIZOFRENIA SIMPLE? | 3 |
| 1 | Garc√≠a-Campayo J | 2007 | UN CASO DE HIKIKOMORI EN ESPA√ëA | 3 |

| | | | | |
|---|---|---|---|---|
| 1 | Burkley M | 2009 | THE POSITIVE (AND NEGATIVE) CONSEQUENCES OF ENDORSING NEGATIVE SELF-STEREOTYPES @ SELF AND IDENTITY | 4 |
| 1 | Avendal C | 2011 | SOCIAL WORK IN GHANA | 4 |
| 1 | Buren PV | 2015 | RETRIEVED FROM @ WHY WON,ÄôT A MILLION JAPANESE LEAVE THEIR HOMES? | 4 |
| 1 | Amichai-Hamburger Y | 2008 | E-EMPOWERMENT | 4 |
| 1 | Aguglia E | 2010 | HIKIKOMORI PHENOMENON | 4 |
| 1 | Biggs BK | 2012 | SOCIAL ANXIETY AND ADOLESCENTS,Äô FRIENDSHIPS | 4 |
| 1 | Borovoy A | 2008 | JAPAN,ÄôS HIDDEN YOUTHS | 4 |
| 2 | Chan GHY | 2014 | DO FRIENDSHIP AND INTIMACY IN VIRTUAL COMMUNICATIONS EXIST? | 4 |
| 1 | Chan GHY | 2010 | @ HIKIKOMORI AND THE INTERNET - EMPOWERMENT AND DISEMPOWERMENT | 4 |
| 2 | Malagv≥n-Amor vÅ | 2014 | HIKIKOMORI IN SPAIN | 4 |
| 2 | Uchida C | 2010 | APATHETIC AND WITHDRAWING STUDENTS IN JAPANESE UNIVERSITIES - WITH REGARD TO HIKIKOMORI AND STUDENT APATHY @ JOURNAL OF MEDICAL AND DENTAL SCIENCES | 4 |
| 4 | Chan GHY | 2014 | HIDDEN YOUTH SERVICES | 4 |
| 1 | Chan GHY | 2014 | FAMILY RELATIONSHIPS AND THE SELF-ESTEEM OF HIDDEN YOUTH | 4 |
| 1 | Baek SB | 2014 | PSYCHOPATHOLOGY OF SOCIAL ISOLATION @ JOURNAL OF EXERCISE REHABILITATION | 4 |
| 1 | Chan GHY | 2013 | QUALITY OF LIFE OF THE HIDDEN YOUTH IN HONG KONG @ APPLIED RESEARCH IN QUALITY OF LIFE | 4 |
| 1 | Chong S | 2012 | A CASE STUDY OF A CHINESE HIKIKOMORIAN IN CANADA - THEORIZING THE PROCESS OF HIKIKOMORIZATION @ JOURNAL OF SPECIAL EDUCATION AND REHABILITATION | 4 |

| | | | | |
|---|---|---|---|---|
| 1 | Funakoshi A | 2014 | SIGNIFICANT FACTORS IN FAMILY DIFFICULTIES FOR FATHERS AND MOTHERS WHO USE SUPPORT SERVICES FOR CHILDREN WITH HIKIKOMORI @ PSYCHIATRY AND CLINICAL NEUROSCIENCES | 4 |
| 4 | Koyama A | 2010 | LIFETIME PREVALENCE, PSYCHIATRIC COMORBIDITY AND DEMOGRAPHIC CORRELATES OF HIKIKOMORI IN A COMMUNITY POPULATION IN JAPAN @ PSYCHIATRY RESEARCH | 4 |
| 2 | Norasakkunkit V | 2014 | TO CONFORM OR TO MAINTAIN SELF-CONSISTENCY? | 4 |
| 1 | Chan GHY | 2014 | HIDDEN YOUTH IN HONG KONG, NEGATIVE EMOTIONS, AND DEVIANT BEHAVIOR @ ISSUES ON JUVENILE CRIMES AND DELINQUENCY | 4 |
| 1 | Furuhashi T | 2013 | √âTAT DES LIEUX, POINTS COMMUNS ET DIFF√âRENCES ENTRE DES JEUNES ADULTES RETIRANTS SOCIAUX EN FRANCE ET AU JAPON (HIKIKOMORI) CURRENT SITUATION, COMMONALITIES AND DIFFERENCES BETWEEN SOCIALLY WITHDRAWN YOUNG ADULTS (HIKIKOMORI) IN FRANCE AND JAPAN @ EVOL PSYCHIATR (PARIS) | 4 |
| 1 | Asakura S | 2012 | SOCIAL ANXIETY TAIJIN-KY≈åFU SCALE (SATS) | 4 |
| 4 | Lee YS | 2013 | HOME VISITATION PROGRAM FOR DETECTING, EVALUATING AND TREATING SOCIALLY WITHDRAWN YOUTH IN KOREA @ PSYCHIATRY AND CLINICAL NEUROSCIENCES | 4 |
| 1 | Bhugra D | 2007 | TEXTBOOK OF CULTURAL PSYCHIATRY | 4 |
| 1 | Chan GHY | 2013 | APPLIED RESEARCH IN QUALITY OF LIFE | 4 |
| 1 | Essau CA | 2011 | A JAPANESE FORM OF SOCIAL ANXIETY TAIJIN KY≈åFUSH≈å | 4 |
| 1 | Chan GHY | 2014 | HIDDEN YOUTH IN HONG KONG, NEGATIVE EMOTIONS, AND DELINQUENT BEHAVIOR @ ISSUES ON JUVENILE CRIMES AND DELINQUENCY | 4 |
| 3 | Garcia-Campayo J | 2007 | A CASE REPORT OF HIKIKOMORI IN SPAIN @ MED CLIN (BARC) | 4 |
| 1 | Fansten M | 2014 | PARIS @ @ HIKIKOMORI | 4 |
| 1 | Chen YW | 2009 | SINGAPORE @ @ ONCE ‚ÄúNEET | 4 |
| 1 | Chan GHY | 2013 | JOURNAL OF FAMILY ISSUES | 4 |

| | | | | |
|---|---|---|---|---|
| 1 | Essau CA | 2012 | TAIJIN KY≈åFUSH≈å AND SOCIAL PHOBIA SYMPTOMS IN YOUNG ADULTS IN ENGLAND AND IN JAPAN @ J CROSS CULT PSYCHOL | 4 |
| 1 | Chen YE | 2014 | @ SOCIAL WORK IN YEAR 104 | 4 |
| 1 | Chan GHY | 2013 | @ HIDDEN YOUTH IN HONG KONG - IN SOCIAL CENSURE PERSPECTIVE | 4 |
| 2 | Umeda M | 2012 | ASSOCIATION OF CHILDHOOD FAMILY ENVIRONMENTS WITH THE RISK OF SOCIAL WITHDRAWAL (HIKIKOMORI) IN THE COMMUNITY POPULATION IN JAPAN @ PSYCHIATRY AND CLINICAL NEUROSCIENCES | 4 |
| 1 | Chong SSC | 2012 | A CASE STUDY OF A CHINESE ‚ÄúHIKIKOMORIAN | 4 |
| 1 | Dziesinski MJ | 2009 | @ FROM FAILED SONS TO WORKING MEN | 4 |
| 1 | De Michele F | 2013 | HIKIKOMORI | 4 |
| 3 | Wong V | 2009 | YOUTH LOCKED IN TIME AND SPACE? | 4 |
| 1 | Bor W | 2014 | ARE CHILD AND ADOLESCENT MENTAL HEALTH PROBLEMS INCREASING IN THE 21ST CENTURY? | 4 |
| 1 | Can culture create mental disease? | 2012 | @ THE RISE OF "HIKIKOMORI" IN THE WAKE OF ECONOMIC DOWNTURN IN JAPAN | 4 |
| 1 | Carli V | 2014 | A NEWLY IDENTIFIED GROUP OF ADOLESCENTS AT "INVISIBLE" RISK FOR PSYCHOPATHOLOGY AND SUICIDAL BEHAVIOR | 4 |
| 3 | Toivonen T | 2011 | UNABLE TO CONFORM, UNWILLING TO REBEL? | 4 |
| 6 | Borovoy A | 2008 | JAPANS HIDDEN YOUTHS | 7 |
| 1 | Carpe Fidem | 2013 | RETRIEVED JULY 5 2013 FROM HTTP://WWW.CARPEFIDEM.COM | 7 |
| 1 | e-Research & Solutions | 2014 | A RESEARCH REPORT ON HIDDEN YOUTH IN MACAU | 7 |

| | | | | |
|---|---|---|---|---|
| 1 | Chinese Evangelical Zion Church Social Service Division | 2013 | Animal attraction — Creature comforts Retrieved July 5, 2013 from http://www.hkzion.org.hk/service.asp?page=%E6%9C%8D%E5%8B%99%E4%BB%8B%E7%B4%B9&sub-page=%E9%87%8D%E6%8B%BE%E5%8B%95%E5%8A%9B | 7 |
| 1 | Caritas Hong Kong | 2013 | BLUE SKY PROGRAMME Retrieved July 5, 2013, from http://lmsit.caritas.org.hk/blue.htm | 7 |
| 1 | ASIAN PUBLIC INTELLECTUALS FELLOWSHIPS | 2009 | CONFLUENCES AND CHALLENGES IN BUILDING THE ASIAN COMMUNITY IN THE EARLY 21ST CENTURY: THE WORK OF THE 2008/2009 API FELLOWS | 7 |
| 1 | Barnes SH | 2011 | @ VOICE OF JAPANESE HIKIKOMORI (SOCIAL WITHDRAWAL) PAPER PRESENTED AT THE NATIONAL COMMUNICATION ASSOCIATION CONFERENCE | 7 |
| 1 | The Community Chest | 2013 | BOCHK “Caring Hong Kong — A Heart Warming Campaign” Retrieved July 5, 2013 from http://www.commchest.org/hk/member/member_01_01.aspx | 7 |
| 5 | Garcia-Campayo J | 2007 | A CASE REPORT OF HIKIKOMORI IN SPAIN @ MEDICINA CLINICA | 7 |
| 1 | Grossman K | 2005 | @ ATTACHMENT FROM INFANCY TO ADULTHOOD | 7 |
| 1 | Hattori Y | 2005 | SOCIAL WITHDRAWAL IN JAPANESE YOUTH | 7 |
| 1 | Booth-Laforce C | 2008 | TRAJECTORIES OF SOCIAL WITHDRAWAL FROM GRADES 1 TO 6 | 7 |
| 1 | Aoki S | 2010 | GOVERNMENT ESTIMATES 700,000 HIKIKOMORI AND 1.5 MILLION AT RISK | 7 |
| 1 | Cunha M | 2008 | THE ROLE OF INDIVIDUAL TEMPERAMENT, FAMILY AND PEERS IN SOCIAL ANXIETY DISORDER | 7 |

| | | | | |
|---|---|---|---|---|
| 1 | Hastings PD | 2010 | @ THE DEVELOPMENT OF SHYNESS AND SOCIAL WITHDRAWAL | 7 |
| 1 | Friedlmeier W | 2006 | ATTACHMENT TRANSFER AMONG GERMAN AND SWEDISH ADOLESCENTS | 7 |
| 1 | Bosquet M | 2006 | THE DEVELOPMENT AND MAINTENANCE OF ANXIETY SYMPTOMS FROM INFANCY THROUGH ADOLESCENCE IN A LONGITUDINAL SAMPLE @ DEVELOPMENT AND PSYCHOPATHOLOGY | 7 |
| 1 | Granqvist P | 2006 | ATTACHMENT AND SPIRITUAL DEVELOPMENT IN CHILDHOOD AND ADOLESCENCE | 7 |
| 1 | MINISTRY OF HEALTH, LABOR & WELFARE | 2013 | The project for promoting measures for hikikomori (2013) Retrieved July 19, 2013 from http://www.mhlw.go.jp/seisakunitsuite/bunya/hukushi_kaigo/seikatsuhogo/hikikomori/index.html | 7 |
| 1 | Chan G | 2010 | @ HIKIKOMORI AND THE INTERNET - EMPOWERMENT AND DISEMPOWERMENT | 7 |
| 1 | Dziesinski MJ | 2007 | HIKIKOMORI: INVESTIGATIONS INTO THE PHENOMENON OF ACUTE SOCIAL WITHDRAWAL IN CONTEMPORARY JAPAN HTTP://TOWAKUDAIBLOGSCOM/HIKIKOMORIRESEARCHSURVEYPDF RETRIEVED JUNE 10 2007 FROM | 7 |
| 1 | Chong SSC | 2012 | A CASE STUDY OF A CHINESE "HIKIKOMORIAN" IN CANADA - THEORIZING THE PROCESS OF HIKIKOMORIZATION @ JOURNAL OF SPECIAL EDUCATION AND REHABILITATION | 7 |
| 1 | Furlong A | 2007 | SUPPORTING THE TRANSITIONS OF VULNERABLE YOUTH | 7 |
| 1 | Gardner M | 2008 | ADOLESCENTS PARTICIPATION IN ORGANIZED ACTIVITIES AND DEVELOPMENTAL SUCCESS 2 AND 8YEARS AFTER HIGH SCHOOL | 7 |
| 1 | Chan G | 2013 | APPLIED RESEARCH IN QUALITY OF LIFE | 7 |
| 1 | Gozlan M | 2012 | 2013 FROM @ THE GROWING BAND OF YOUNG PEOPLE LIVING A RECLUSIVE LIFE @ GUARDIAN WEEKLY | 7 |
| 1 | Chan G | 2014 | FAMILY RELATIONSHIPS AND THE SELF-ESTEEM OF THE HIDDEN YOUTH @ JOURNAL OF FAMILY ISSUES | 7 |

| | | | | |
|---|---|---|---|---|
| 1 | Dziesinski MJ | 2013 | FROM FAILED SONS TO WORKING MEN: REHABILITATING HIKIKOMORI HTTP://SOCIALSCIENCESPEOPLEHAWAIIEDU/PUBLICATIONS | 7 |
| 1 | Chen YW | 2009 | SINGAPORE @ ONCE "NEET", ALWAYS "NEET"? | 7 |
| 1 | Corrigan PW | 2007 | HOW CLINICAL DIAGNOSIS MIGHT EXACERBATE THE STIGMA OF MENTAL ILLNESS @ SOCIAL WORK | 7 |
| 1 | Chan G | 2013 | QUALITY OF LIFE OF THE HIDDEN YOUTH IN HONG KONG @ APPLIED RESEARCH IN QUALITY OF LIFE | 7 |
| 1 | Fu KW | 2010 | INTERNET ADDICTION | 7 |
| 4 | Kato TA | 2018 | HIKIKOMORI | 8 |
| 2 | Kwon M | 2013 | THE SMARTPHONE ADDICTION SCALE | 8 |
| 1 | Bae SM | 2017 | SMARTPHONE ADDICTION OF ADOLESCENTS, NOT A SMART CHOICE @ J KOREAN MED SCI | 8 |
| 1 | Bingham CR | 2015 | DO AS I SAY, NOT AS I DO | 8 |
| 1 | Baer S | 2011 | GREEN, DA STUCK ON SCREENS | 8 |
| 1 | Biolcati R | 2017 | PRONESS TO BOREDOM AND RISK BEHAVIORS DURING ADOLESCENTS' FREE TIME | 8 |
| 1 | Bhagat S | 2015 | IS FACEBOOK A PLANET OF LONELY INDIVIDUALS? | 8 |
| 1 | Bagot KS | 2015 | ADOLESCENT INITIATION OF CANNABIS USE AND EARLY ONSET PSYCHOSIS @ SUBST ABUSE | 8 |
| 1 | Arquero JL | 2015 | VOCATION, MOTIVATION AND APPROACHES TO LEARNING | 8 |
| 1 | Bao Z | 2018 | SCHOOL CONNECTEDNESS AND CHINESE ADOLESCENTS SLEEP PROBLEMS | 8 |
| 1 | Altman NG | 2012 | SLEEP DURATION VERSUS SLEEP INSUFFICIENCY AS PREDICTORS OF CARDIOMETABOLIC HEALTH OUTCOMES @ SLEEP MED | 8 |
| 2 | Kuss DJ | 2014 | INTERNET ADDICTION | 8 |
| 1 | Bland JM | 2015 | STATISTICS NOTES | 8 |
| 1 | Beard CL | 2017 | AGE OF INITIATION AND INTERNET GAMING DISORDER | 8 |
| 1 | Anderson EL | 2017 | INTERNET USE AND PROBLEMATIC INTERNET USE | 8 |

| | | | | |
|---|---|---|---|---|
| 1 | American Psychiatric Association | 2013 | DIAGNOSTIC AND STATISTICAL MANUAL OF MENTAL DISORDERS | 8 |
| 1 | Beard CL | 2016 | GAMING-CONTINGENT SELF-WORTH, GAMING MOTIVATION, AND INTERNET GAMING DISORDER @ COMPUTERS IN HUMAN BEHAVIOR | 8 |
| 1 | Arnett JJ | 2014 | THE NEW LIFE STAGE OF EMERGING ADULTHOOD AT AGES 18,Äì29,ÄØYEARS | 8 |
| 1 | Adams BL | 2018 | INTERNET GAMING DISORDER BEHAVIORS IN EMERGENT ADULTHOOD | 8 |
| 1 | Kato TA | 2019 | DEVELOPMENT AND VALIDATION OF THE 22-ITEM TARUMIS MODERN-TYPE DEPRESSION TRAIT SCALE | 8 |
| 2 | Kato TA | 2016 | MULTIDIMENSIONAL ANATOMY OF MODERN TYPE DEPRESSION IN JAPAN | 8 |
| 1 | Andreassen CS | 2012 | DEVELOPMENT OF A FACEBOOK ADDICTION SCALE @ PSYCHOL REP | 8 |
| 3 | Kato TA | 2012 | DOES THE HIKIKOMORI SYNDROME OF SOCIAL WITHDRAWAL EXIST OUTSIDE JAPAN? | 8 |
| 1 | Bhugra D | 2017 | THE WPA-LANCET PSYCHIATRYCOMMISSION ON THE FUTURE OF PSYCHIATRY @ THE LANCET PSYCHIATRY | 8 |
| 4 | Wong PW | 2015 | THE PREVALENCE AND CORRELATES OF SEVERE SOCIAL WITHDRAWAL (HIKIKOMORI) IN HONG KONG | 8 |
| 2 | Umeda M | 2012 | ASSOCIATION OF CHILDHOOD FAMILY ENVIRONMENTS WITH THE RISK OF SOCIAL WITHDRAWAL (,ÄòHIKIKOMORI,Äô) IN THE COMMUNITY POPULATION IN JAPAN @ PSYCHIATRY AND CLINICAL NEUROSCIENCES | 8 |
| 4 | Chauliac N | 2017 | CHARACTERISTICS OF SOCIALLY WITHDRAWN YOUTH IN FRANCE | 8 |
| 4 | Kato TA | 2011 | ARE JAPANS HIKIKOMORI AND DEPRESSION IN YOUNG PEOPLE SPREADING ABROAD? | 8 |
| 3 | Azuma H | 2009 | @ OTAKU | 9 |
| 3 | Brinton M | 2011 | @ LOST IN TRANSITION | 9 |
| 1 | Goodman R | 2006 | POLICING THE JAPANESE FAMILY | 9 |
| 1 | Azuma H | 2008 | TO BE PRIVATELY PUBLIC | 9 |

| | | | | |
|---|---|---|---|---|
| 1 | Horiguchi S | 2012 | ABINGDON | 9 |
| 1 | Furuichi N | 2011 | K≈åDANSHA @ @ THE HAPPY YOUTH OF A DESPERATE COUNTRY | 9 |
| 1 | Eng L | 2009 | @ THE CURRENT STATUS OF ,ÄúOTAKU,Äù AND JAPAN,ÄôS LATEST YOUTH CRISIS | 9 |
| 1 | Best J | 2013 | ASHGATE @ THE PROBLEMS WITH MORAL PANIC | 9 |
| 1 | Fu H | 2011 | @ AN EMERGING NON-REGULAR LABOUR FORCE IN JAPAN | 9 |
| 1 | Ambaras D | 2006 | @ BAD YOUTH | 9 |
| 1 | Galbraith P | 2009 | MOE | 9 |
| 1 | Honda Y | 2005 | TOKYO UNIVERSITY PRESS @ @ YOUTH AND WORK | 9 |
| 1 | Allison A | 2009 | THE AFFECTIVE ACTIVISM OF JAPANESE YOUTH @ THEORY | 9 |
| 1 | Borovoy A | 2008 | JAPAN,ÄôS HIDDEN YOUTHS | 9 |
| 1 | Chiavacci D | 2008 | FROM CLASS STRUGGLE TO GENERAL MIDDLE-CLASS SOCIETY TO DIVIDED SOCIETY | 9 |
| 2 | Borovoy A | 2008 | JAPAN,ÄôS HIDDEN YOUTHS | 9 |
| 1 | Azuma H | 2010 | @ NIHONTEKI S≈åZ≈åRYOKU NO MIRAI | 9 |
| 1 | Casanova P | 2004 | HARVARD UNIVERSITY PRESS @ @ THE WORLD REPUBLIC OF LETTERS | 9 |
| 1 | Azuma H | 2007 | @ GƒíMU-TEKI RIARIZUMU NO TANJ≈å | 9 |
| 1 | Harney A | 2009 | @ JAPAN PANICS ABOUT THE RISE OF ,ÄòHERBIVORES,Äô,ÄìYOUNG MEN WHO SHUN SEX | 9 |
| 1 | Backhaus J | 2013 | ALGONQUIN @ @ HIKIKOMORI AND THE RENTAL SISTER | 9 |
| 1 | Carla R | 2010 | @ HIKIKOMORI | 9 |
| 1 | HTTP://WWW8.CAO.GO.JP/YOUTH/KENKYU/HIKIKOMORI/PDF/S3.PDF | 2011 | EMPIRICAL SURVEY ON HIKIKOMORI | 9 |
| 1 | Arnett JJ | 2011 | EMERGING ADULTHOOD(S) | 9 |

| | | | | |
|---|---|---|---|---|
| 1 | Bellah ERN | 2007 | UNIVERSITY OF CALIFORNIA PRESS @ @ HABITS OF THE HEART | 9 |
| 5 | Allison A | 2013 | @ PRECARIOUS JAPAN | 11 |
| 2 | ARLINGTON (5TH ED) | 2013 | DIAGNOSTIC AND STATISTICAL MANUAL OF MENTAL DISORDERS | 11 |
| 1 | Caputo A | 2014 | THE SOCIAL CONSTRUCTION OF ENVY IN SCIENTIFIC COMMUNITY | 11 |
| 1 | Caputo A | 2015 | TRENDS OF PSYCHOLOGY-RELATED RESEARCH ON EUTHANASIA | 11 |
| 1 | Caputo A | 2013 | HEALTH DEMAND IN PRIMARY CARE CONTEXT | 11 |
| 1 | Caputo A | 2013 | CULTURAL MODELS SHAPING STALKING FROM A CONTENT ANALYSIS OF ITALIAN NEWSPAPERS @ EUROPES JOURNAL OF PSYCHOLOGY | 11 |
| 2 | Pozza A | 2019 | THE HIKIKOMORI SYNDROME | 11 |
| 12 | Kato TA | 2018 | HIKIKOMORI | 11 |
| 2 | Tajan N | 2017 | TRAUMATIC DIMENSIONS OF HIKIKOMORI | 11 |
| 1 | Bovoroy A | 2012 | DOI TAKEO AND THE REHABILITATION OF PARTICULARISM IN POSTWAR JAPAN @ THE JOURNAL OF JAPANESE STUDIES | 11 |
| 1 | Ahmed S | 2014 | @ WILLFUL SUBJECTS | 11 |
| 1 | Beran D | 2017 | HOW THE ISOLATED MAN-BOYS OF 4CHAN TURNED A MEME INTO THE PRESIDENT OF THE UNITED STATES | 11 |
| 1 | Berlant L | 2011 | @ CRUEL OPTIMISM | 11 |
| 1 | Ahmed S | 2010 | @ THE PROMISE OF HAPPINESS | 11 |
| 1 | Aaltonen S | 2015 | NUORET LUUKULLA | 11 |
| 1 | Aaltonen S | 2019 | TY√ñN JA KOULUTUKSEN ULKOPUOLELLA OLEVIEN NUORTEN AIKUISTEN KOETTU HYVINVOINTI @ YHTEISKUNTAPOLITIIKKA | 11 |
| 1 | Bowker J | 2016 | ENCYCLOPAEDIA OF ADOLESCENCE | 11 |
| 1 | Davis EA | 2012 | @ BAD SOULS | 11 |
| 1 | Behrens KY | 2011 | JAPANESE CHILDRENS AMAE AND MOTHERS ATTACHMENT STATUS AS ASSESSED BY THE ADULT ATTACHMENT INTERVIEW @ INTERNATIONAL JOURNAL OF PSYCHOLOGY | 11 |
| 1 | Cook EE | 2013 | EXPECTATIONS OF FAILURE | 11 |
| 2 | Rooksby M | 2020 | HIKIKOMORI | 11 |

| | | | | |
|---|---|---|---|---|
| 1 | Am√©d√©e LM | 2019 | CHILD VICTIMS OF SEXUAL ABUSE | 11 |
| 1 | Al-Adawi S | 2001 | ZAR | 15 |
| 1 | Al-Adawi S | 1998 | MOTIVATIONAL DEFICITS AFTER BRAIN INJURY | 15 |
| 1 | Campbell SS | 1999 | ETIOLOGY AND TREATMENT OF INTRINSIC CIRCADIAN RHYTHM SLEEP DISORDERS @ SLEEP MEDICINE REVIEWS | 15 |
| 1 | Haeri N | 2003 | @ SACRED LANGUAGE | 15 |
| 2 | Murakami R | 2000 | @ SYMBIOSIS WORM KYOSEICHU | 15 |
| 3 | Kawanishi Y | 2004 | JAPANESE YOUTH | 15 |
| 1 | Koenig HG | 2000 | MEDICINE AND RELIGION @ NEW ENGLAND JOURNAL OF MEDICINE | 15 |
| 1 | Torgersen S | 2001 | THE PREVALENCE OF PERSONALITY DISORDERS IN A COMMUNITY SAMPLE @ ARCHIVES OF GENERAL PSYCHIATRY | 15 |
| 1 | Neal JA | 2003 | THE ETIOLOGY OF SOCIAL PHOBIA | 15 |
| 1 | Tamaki S | 1998 | @ SOCIAL WITHDRAWAL | 15 |
| 1 | Watanabe T | 2003 | SLEEP AND CIRCADIAN RHYTHM DISTURBANCES IN PATIENTS WITH DELAYED SLEEP PHASE SYNDROME @ SLEEP | 15 |
| 1 | WASHINGTON: AMERICAN PSYCHIATRIC ASSOCIATION TEXT REV | 2000 | DIAGNOSTIC AND STATISTICAL MANUAL OF MENTAL DISORDERS IV-TR. 4 ED. | 15 |
| 1 | Watts J | 2002 | PUBLIC HEALTH EXPERTS CONCERNED ABOUT "HIKIKOMORI | 15 |
| 1 | Tyrer P | 2002 | NIDOTHERAPY | 15 |
| 2 | Alarcon RD | 2002 | BEYOND THE FUNHOUSE MIRRORS @ A RESEARCH AGENDA FOR DSM-V | 15 |

| | | | | |
|---|---|---|---|---|
| 1 | Sakamoto N | 2005 | HIKIKOMORI, IS IT A CULTURE-REACTIVE OR CULTURE-BOUND SUYNDROME? | 15 |
| 1 | Watts J | 2002 | PUBLIC HEALTH EXPERTS CONCERNED ABOUT HIKIKOMORI @ LANCET | 15 |
| 3 | Berardi F | 2015 | @ HEROES | 16 |
| 12 | Saito T | 2013 | @ HIKIKOMORI | 16 |
| 1 | Berardi F | 2020 | BEYOND THE BREAKDOWN | 16 |
| 1 | Armitage J | 2013 | AESTHETICS, VISION AND SPEED | 16 |
| 1 | Auerbach D | 2012 | FEBRUARY @ ANONYMITY AS CULTURE | 16 |
| 1 | Berardi F | 2014 | @ PHENOMENOLOGY OF THE END | 16 |
| 1 | Berardi F | 2020 | RESPIRACION UMBRAL | 16 |
| 1 | Armitage J | 2011 | PAUL VIRILIO | 16 |
| 1 | Athique A | 2019 | ADVANCE ONLINE PUBLICATION @ INTEGRATED COMMODITIES IN THE DIGITAL ECONOMY @ MEDIA | 16 |
| 1 | Beran D | 2017 | FEBRUARY @ FROM FAIL TO WIN | 16 |
| 1 | Berardi F | 2021 | @ THE THIRD UNCONSCIOUS | 16 |
| 1 | Bonneuil C | 2017 | @ THE SHOCK OF THE ANTHROPOCENE | 16 |
| 2 | Teo A | 2015 | IDENTIFICATION OF THE HIKIKOMORI SYNDROME OF SOCIAL WITHDRAWAL | 16 |
| 1 | Adhanom Ghebreyesus T | 2020 | @ WORLD PSYCHIATRY | 16 |
| 1 | Cacioppo JT | 2015 | LONELINESS ACROSS PHYLOGENY AND A CALL FOR COMPARATIVE STUDIES AND ANIMAL MODELS @ PERSPECTIVES ON PSYCHOLOGICAL SCIENCE | 16 |
| 2 | Teo AR | 2015 | IDENTIFICATION OF THE HIKIKOMORI SYNDROME OF SOCIAL WITHDRAWAL | 16 |
| 7 | Tajan N | 2015 | SOCIAL WITHDRAWAL AND PSYCHIATRY | 17 |
| 2 | Teo AR | 2010 | HIKIKOMORI, A JAPANESE CULTURE-BOUND SYNDROMEOF SOCIAL WITHDRAWAL? | 17 |
| 2 | Kato T | 2012 | DOES HIKIKOMORI SYNDROME OF SOCIAL WITHDRAWAL EXIST OUTSIDE JAPAN? | 17 |

| | | | | |
|---|---|---|---|---|
| 2 | Komaya A | 2010 | LIFETIME PREVALENCE, PSYCHIATRIC COMORBIDITY AND DEMOGRAPHIC CORRELATES OF ‚ÄúHIKIKOMORI‚Äù IN A COMMUNITY POPULATION IN JAPAN @ PSYCHIATR RES | 17 |
| 2 | Kondo N<br>Sakai M<br>Kuroda Y<br>Kiyota Y<br>Kitabata Y<br>Kurosawa M | 2013 | GENERAL CONDITION OF HIKIKOMORI (PROLONGED SOCIAL WITHDRAWAL) IN JAPAN | 17 |
| 2 | Krieg A<br>Dickie JR | 2013 | ATTACHMENT AND HIKIKOMORI | 17 |
| 2 | De Luca M | 2013 | LE HIKIKOMORI √Ä LADOLESCENCE | 17 |
| 2 | Furuashi T | 2013 | √âTAT DES LIEUX, POINTS COMMUNS ET DIFF√âRENCES ENTRE DES JEUNES ADULTES RETIRANTS SOCIAUX EN FRANCE ET AU JAPON (HIKIKOMORI) @ EVOL PSYCHIATR | 17 |
| 1 | Arimitsu K | 2014 | DEVELOPMENT AND VALIDATION OF THE JAPANESE VERSION OF THE SELF-COMPASSION SCALE @ SHINRIGAKU KENKYU | 17 |
| 1 | Ito J | 2021 | GUIDELINE ON MENTAL HEALTH ACTIVITIES IN COMMUNITIES FOR SOCIAL WITHDRAWAL | 17 |

| | | | | |
|---|---|---|---|---|
| 1 | UFFICIO SCOLASTICO REGIONALE PER LEMILIA-ROMAGNA MINISTERO DELLISTRUZIONE | 2020 | ADOLESCENTI ,ÄúEREMITI SOCIALI,Äù RILEVAZIONE NELLE SCUOLE DELLEMILIA-ROMAGNA DEGLI ALUNNI CHE NON FREQUENTANO ,ÄúRITIRATI,Äù IN CASA PER MOTIVI PSICOLOGICI HTTPS://BITLY/3RC7KTJ UFFICIO SCOLASTICO REGIONALE PER LEMILIA-ROMAGNA MINISTERO DELLISTRUZIONE DISPONIBILE SU: ULTIMO ACCESSO 11 MARZO 2020 | 17 |
| 1 | Arimitsu K Aoki Y Furukita M Tada A Togashi R | 2016 | CONSTRUCTION AND VALIDATION OF A SHORT FORM OF THE JAPANESE VERSION OF THE SELF-COMPAS-SION SCALE @ KOMAZAWA ANN REP PSYCH | 17 |
| 1 | UFFICIO SCOLASTICO REGIONALE PER LEMILIA-ROMAGNA MINISTERO DELLISTRUZIONE | 2020 | ALUNNI PER ANNO DI CORSO GENERE CITTADINANZA E COMUNE AS 2018/19 - DATI AGGIORNATI AL 19-03-2019 HTTPS://BITLY/3RGISL8 UFFICIO SCOLASTICO REGIONALE PER LEMILIA-ROMAGNA MINISTERO DELLISTRUZIONE DISPONIBILE SU: ULTIMO ACCESSO 11 MARZO 2020 | 17 |

| | | | | |
|---|---|---|---|---|
| 4 | Stip E Thibault A Beauchamp-Chatel A Kisely S | 2016 | INTERNET ADDICTION, HIKIKOMORI SYNDROME, AND THE PRODROMAL PHASE OF PSYCHOSIS @ FRONT PSYCHIATRY | 17 |
| 1 | Becedas L√≥pez A Aragon√©s Callejas M Eguiluz Fern√°ndez M Sotomayor S√°ez MA Fern√°ndez Montero R | 2018 | RELEVANCIA CL√çNICA DEL ABORDAJE ENFERMERO A ADOLESCENTES EN DOMICILIO | 17 |
| 1 | Beaven Ciapara NI Campa √Ålvarez RA Valenzuela BA Guill√©n L√∫gigo M | 2018 | INCLUSI√ìN EDUCATIVA | 17 |
| 15 | Tateno M | 2012 | HIKIKOMORI AS A POSSIBLE CLINICAL TERM IN PSYCHIATRY | 28 |
| 1 | Best JR | 2013 | EXERGAMING IN YOUTH @ ZEITSCHRIFT F√úR PSYCHOLOGIE | 28 |
| 1 | Baranowski T | 2016 | GAMES FOR HEALTH FOR CHILDREN | 28 |
| 1 | Anderson N | 2017 | POKEMON GO | 28 |

| | | | | |
|---|---|---|---|---|
| 1 | Buddharaju P | 2016 | PROCEEDINGS OF THE INTERNATIONAL WORKSHOP ON MOBILE SOFTWARE ENGINEERING AND SYSTEMS - MOBILESOFT‚Äô16 | 28 |
| 1 | Cao R | 2016 | @ HOW ‚ÄòPOKEMON GO‚Äô HELPS KIDS WITH AUTISM AND ASPERGER‚ÄôS - CNNCOM | 28 |
| 1 | Althoff T | 2016 | INFLUENCE OF POK√âMON GO ON PHYSICAL ACTIVITY | 28 |
| 1 | Anderson-Hanley C | 2011 | AUTISM AND EXERGAMING | 28 |
| 2 | Tateno M | 2016 | NEW GAME SOFTWARE (POKEMON GO) MAY HELP YOUTH WITH SEVERE SOCIAL WITHDRAWAL, HIKIKOMORI @ PSYCHIATRY RES | 28 |
| 1 | Blom RM | 2011 | ASSOCIATION BETWEEN A SEROTONIN TRANSPORTER PROMOTER POLYMORPHISM (5HTTLPR) AND PERSONALITY DISORDER TRAITS IN A COMMUNITY SAMPLE @ J PSYCHIATR RES | 29 |
| 1 | Arora V | 2013 | POSSIBLE INVOLVEMENT OF OXIDO-NITROSATIVE STRESS INDUCED NEURO-INFLAMMATORY CASCADE AND MONOAMINERGIC PATHWAY | 29 |
| 1 | Brugha TS | 2011 | EPIDEMIOLOGY OF AUTISM SPECTRUM DISORDERS IN ADULTS IN THE COMMUNITY IN ENGLAND @ ARCH GEN PSYCHIATRY | 29 |
| 1 | Camont L | 2011 | BIOLOGICAL ACTIVITIES OF HDL SUBPOPULATIONS AND THEIR RELEVANCE TO CARDIOVASCULAR DISEASE @ TRENDS MOL MED | 29 |
| 1 | Brugha TS | 2016 | EPIDEMIOLOGY OF AUTISM IN ADULTS ACROSS AGE GROUPS AND ABILITY LEVELS @ BR J PSYCHIATRY | 29 |
| 1 | Capuron L | 2011 | RELATIONSHIP BETWEEN ADIPOSITY, EMOTIONAL STATUS AND EATING BEHAVIOUR IN OBESE WOMEN | 29 |
| 1 | Burton-Chellew MN | 2012 | PSEUDOCOMPETITION AMONG GROUPS INCREASES HUMAN COOPERATION IN A PUBLIC-GOODS GAME @ ANIMAL BEHAVIOUR | 29 |
| 1 | Black CN | 2017 | URIC ACID IN MAJOR DEPRESSIVE AND ANXIETY DISORDERS @ J AFFECT DISORD | 29 |

| | | | | |
|---|---|---|---|---|
| 1 | Alici D | 2016 | EVALUATION OF OXIDATIVE METABOLISM AND OXIDATIVE DNA DAMAGE IN PATIENTS WITH OBSESSIVE-COMPULSIVE DISORDER @ PSYCHIATRY CLIN NEUROSCI | 29 |
| 1 | Alvarez-Mon MA | 2018 | INCREASING INTEREST OF MASS COMMUNICATION MEDIA AND THE GENERAL PUBLIC IN THE DISTRIBUTION OF TWEETS ABOUT MENTAL DISORDERS | 31 |
| 1 | Arseniev-Koehler A | 2016 | #PROANA | 31 |
| 1 | Berry N | 2017 | #WHYWETWEETMH | 31 |
| 1 | Birnbaum ML | 2017 | A COLLABORATIVE APPROACH TO IDENTIFYING SOCIAL MEDIA MARKERS OF SCHIZOPHRENIA BY EMPLOYING MACHINE LEARNING AND CLINICAL APPRAISALS @ J MED INTERNET RES | 31 |
| 1 | Alcaraz Mateos E | 2017 | Pathology in social media networks @ Recruitment Campaign] Rev Esp Patol | 31 |
| 1 | Booth R | 2018 | YOUTH MENTAL HEALTH SERVICES UTILIZATION RATES AFTER A LARGE-SCALE SOCIAL MEDIA CAMPAIGN | 31 |
| 1 | Bollen J | 2011 | TWITTER MOOD PREDICTS THE STOCK MARKET @ J COMPUT SCI | 31 |
| 4 | Yong R | 2019 | HIKIKOMORI IS MOST ASSOCIATED WITH INTERPERSONAL RELATIONSHIPS, FOLLOWED BY SUICIDE RISKS | 40 |
| 1 | Arbuckle JL | 2016 | @ SPSS AMOS24 USERS GUIDE | 40 |
| 1 | (5TH ED) WASHINGTON AMERICAN PSYCHIATRIC ASSOCIATION (APA) | 2013 | DIAGNOSTIC AND STATISTICAL MANUAL OF MENTAL DISORDERS | 40 |
| 1 | Arnett JJ | 2014 | THE NEW LIFE STAGE OF EMERGING ADULTHOOD AT AGES 18-29 YEARS | 40 |

| | | | | |
|---|---|---|---|---|
| 2 | Tandon R | 2020 | COVID-19 AND MENTAL HEALTH | 44 |
| 1 | Brooks SK | 2020 | THE PSYCHOLOGICAL IMPACT OF QUARANTINE AND HOW TO REDUCE IT: RAPID REVIEW OF THE EVIDENCE | 44 |
| 1 | Bagcchi S | 2020 | STIGMA DURING THE COVID-19 PANDEMIC @ LANCET INFECT DIS | 44 |
| 1 | Brooks SK | 2020 | THE PSYCHOLOGICAL IMPACT OF QUARANTINE AND HOW TO REDUCE IT | 44 |
